# Migration background and COVID-19 related intensive care unit admission and mortality in the Netherlands: A cohort study

**DOI:** 10.1371/journal.pone.0284036

**Published:** 2023-04-05

**Authors:** Gurbey Ocak, Meriem Khairoun, Martine van Stigt Thans, Danielle Meeder, Hazra Moeniralam, Friedo W. Dekker, Marianne C. Verhaar, Willem Jan W. Bos, Karin A. H. Kaasjager

**Affiliations:** 1 Department of Internal Medicine, Sint Antonius Hospital, Nieuwegein, The Netherlands; 2 Department of Nephrology and Hypertension, University Medical Center Utrecht, Utrecht, The Netherlands; 3 Department of Clinical Epidemiology, Leiden University Medical Center, Leiden, The Netherlands; 4 Department of Intensive Care, Sint Antonius Hospital, Nieuwegein, The Netherlands; 5 Department of Internal Medicine, Leiden University Medical Center, Leiden, The Netherlands; 6 Department of Internal Medicine, University Medical Center Utrecht, Utrecht, The Netherlands; Federal University of Acre (UFAC), BRAZIL

## Abstract

**Background:**

Since the beginning of the SARS-CoV-2 pandemic, studies have been reporting inconsistently on migration background as a risk factor for COVID-19 outcomes. The aim of this study was to evaluate the association between migration background and clinical outcomes with COVID-19 in the Netherlands.

**Methods:**

This cohort study included 2,229 adult COVID-19 patients admitted in two Dutch hospitals between February 27, 2020 and March 31, 2021. Odds ratios (ORs) for hospital admission, intensive care unit (ICU) admission and mortality with 95% confidence intervals (CIs) were calculated for non-Western (Moroccan, Turkish, Surinamese or other) persons as compared with Western persons in the general population of the province of Utrecht (the Netherlands) as source population. Furthermore, among hospitalized patients, Hazard ratios (HRs) with 95% CIs for in-hospital mortality and intensive care unit (ICU) admission were calculated using Cox proportional hazard analyses. Hazard ratios were adjusted for age, sex, body mass index, hypertension, Charlson Comorbidity Index, chronic corticosteroid use before admission, income, education and population density to investigate explanatory variables.

**Results:**

Of the 2,229 subjects, 1,707 were of Western origin and 522 were of non-Western origin. There were 313 in-hospital deaths and 503 ICU admissions. As compared with persons with a Western origin in the general population of the province of Utrecht, the ORs for non-Western persons was 1.8 (95% CI 1.7–2.0) for hospitalization, 2.1 (95% CI 1.7–2.5) for ICU admission and 1.3 (95% CI 1.0–1.7) for mortality. Among hospitalized patients, HR for ICU admission was 1.1 (95% CI 0.9–1.4) and 0.9 (95% CI 0.7–1.3) for mortality for non-Western hospitalized persons as compared with hospitalized patients of Western origin after adjustment.

**Conclusion:**

Non-Western persons, including Moroccan, Turkish and Surinamese subjects, had increased risks of hospital admission, ICU admission and COVID-19 related death on a population level. Among hospitalized COVID-19 patients, no association was found between migration background and ICU admission or mortality.

## Introduction

Studies have been reporting inconsistently about the presence of ethnic disparity in terms of COVID-19 severity and outcomes [1–9]. Large COVID-19 cohort studies across different continents reported higher rates of intensive care unit (ICU) admission [1, 2] and in-hospital death [1–4] among ethnic minority groups compared with white persons. In contrast, several other studies with similar study populations in terms of size and ethnic diversity did not find these associations [5–9].

The role of ethnicity has been the subject of research during previous pandemics. During the 2009/2010 H1N1 influenza outbreak, data analyses from across the United Kingdom showed increased mortality rates for people of non-White origin compared to those of White origin [10]. In addition, actively engaging local ethnic populations turned out to be one of the key factors in controlling the Ebola pandemic in West Africa in 2014 [11]. Given these findings, identification of the vulnerable ethnic groups would be essential for the implementation of targeted prevention measures in order to gain optimal control of the COVID-19 pandemic and future pandemics.

So far, the majority of studies on the relationship between migration background and COVID-19 outcomes originates from the United States [12, 13] or the United Kingdom [14]. Only one recent study from the Amsterdam and its neighboring city Almere in the Netherlands investigated the association between migration background and COVID-19 outcomes [15]. Other studies from other Western European countries on the association between migration background and hospital admission, ICU and mortality are lacking. Furthermore, few studies adjusted for both socioeconomic and clinical factors in the association between migration background and COVID-19 outcomes [9, 12–14].

The aim of this study was to investigate the association between migration background and COVID-19 related hospital admission, ICU admission and death on a population level. Furthermore, we examined the relationship between migration background and length of hospital stay ICU admission and in-hospital mortality among patients hospitalized with COVID-19.

## Material and methods

### Study design and population

This observational cohort study was performed among patients hospitalized with COVID-19 in the two largest hospitals in the province of Utrecht in the Netherlands; the Sint Antonius Hospital (non-academic teaching hospital) and the University Medical Center Utrecht (academic hospital). In the Sint Antonius Hospital, patients were included between March 5, 2020 and January 9, 2021. In the University Medical Center Utrecht, the inclusion period was from February 27, 2020 until March 31, 2021.

The study population comprised adult patients aged 18 years or older who had a polymerase chain reaction proven COVID-19 diagnosis. Patients without a Dutch residential address (n = 3) or without any available information on socioeconomic status (n = 1) were excluded. This study was conducted in accordance with the Declaration of Helsinki. The Medical Research Ethics Committee decided that institutional review board approval was not required (University Medical Center Utrecht), as patients were not subjected to interventions. Patient identification numbers were visible to the researchers in order to access electronic medical files. All data were pseudonymized before analysis. Follow-up started at hospitalization date and ended with either death or discharge from hospital.

### Migration background

Migration background was classified according to the categorization used by the Dutch Central Bureau of Statistics [16]. Patients were grouped into ‘Western origin’ (Europe, North America, Oceania, Indonesia, Japan) or ‘non-Western origin’ (Turkey, Africa, South America and Asia). The non-Western group was further disaggregated into patients of Moroccan, Turkish and Surinamese origin, as these patients represent the three largest minority groups in the Netherlands. The remaining non-Western persons were categorized as other non-Western origin. For each patient, medical records were viewed to collect data on birth place to assess migration background. For patients for whom country of birth was unknown, surname or physician-reported information available in the electronic health record was used to indicate the migration background [15]. A person who was born in Africa, Latin America, Asia or Turkey were classified as a non-western background according to the Dutch Central Bureau of Statistics [16].

### Demographic and clinical data

Data on age and sex were collected through medical record review. Clinical explanatory variables included the individual comorbidities scored in the Charlson Comorbidity Index [17]. Furthermore, data on body mass index, hypertension and chronic use of systemic corticosteroids (before admission) were collected. Hypertension was scored present if patients were using any antihypertensive treatment on admission.

Data on socioeconomic status (income, education and population density) was available on environmental-level socioeconomic status and was not available for the individual patients. Using demographic registries by the Dutch Central Bureau of Statistics based on the postal code of the patient’s residential address [18], patient were categorized into tertiles based on the level of minimum income households, the level of education and population density.

### Outcomes

The primary outcome of our study was in-hospital mortality. Secondary outcomes were ICU admission and length of hospital stay.

### Statistical analysis

Continuous variables were reported as medians with interquartile ranges and categorical variables with counts and percentages. Person-days of follow-up were counted from the date of hospitalization to the date of death or hospital discharge. Patients were censored at the end of observation date (January 9, 2021 for the Sint Antonius Hospital and March 31, 2021 for the University Medical Center Utrecht) or when transferred to another hospital.

To investigate the association between migration background and COVID-19 related hospital admission, ICU admission and death on a population level, the distribution of the migration background in the general population of the province of Utrecht (the Netherlands) (reference) were compared with the distribution in patients who were admitted to the hospitals, ICU and patients who died due to COVID-19. For this purpose, odds ratios (ORs) with 95% confidence intervals (CIs) were calculated for COVID-19 related hospital admission, ICU admission and death.

In addition, groups with different migration backgrounds were compared after admission to the hospital. Cox proportional hazard regression models were used to evaluate the association between migration background and ICU admission and in-hospital mortality. Crude hazard ratios (HR) with 95% confidence intervals (CIs) were calculated, with the patient group of Western origin as the reference category. Furthermore, crude and adjusted Beta Coefficients with 95% CIs were calculated using linear regression to investigate the association between migration background and length of hospital stay and ICU stay. To investigate the potential role of explanatory variables in the association between migration background and outcomes (mortality, ICU admission or length of hospital stay), we adjusted the regression analyses in four models. In model 1, HRs were adjusted for age and sex only. In model 2, we also adjusted for body mass index, hypertension, Charlson Comorbidity Index and chronic use of systemic corticosteroids (before admission). In model 3, environmental socioeconomic status (income and education) was added as potential explanatory variable. In the final model, HRs were fully adjusted for age, sex, comorbidities, medication use, income, education and population density. Data analyses were performed using IBM SPSS Statistics for Windows, Version 26.0 (IBM Corp, Armonk, NY, USA).

## Results

### Baseline characteristics

**Fig 1:**
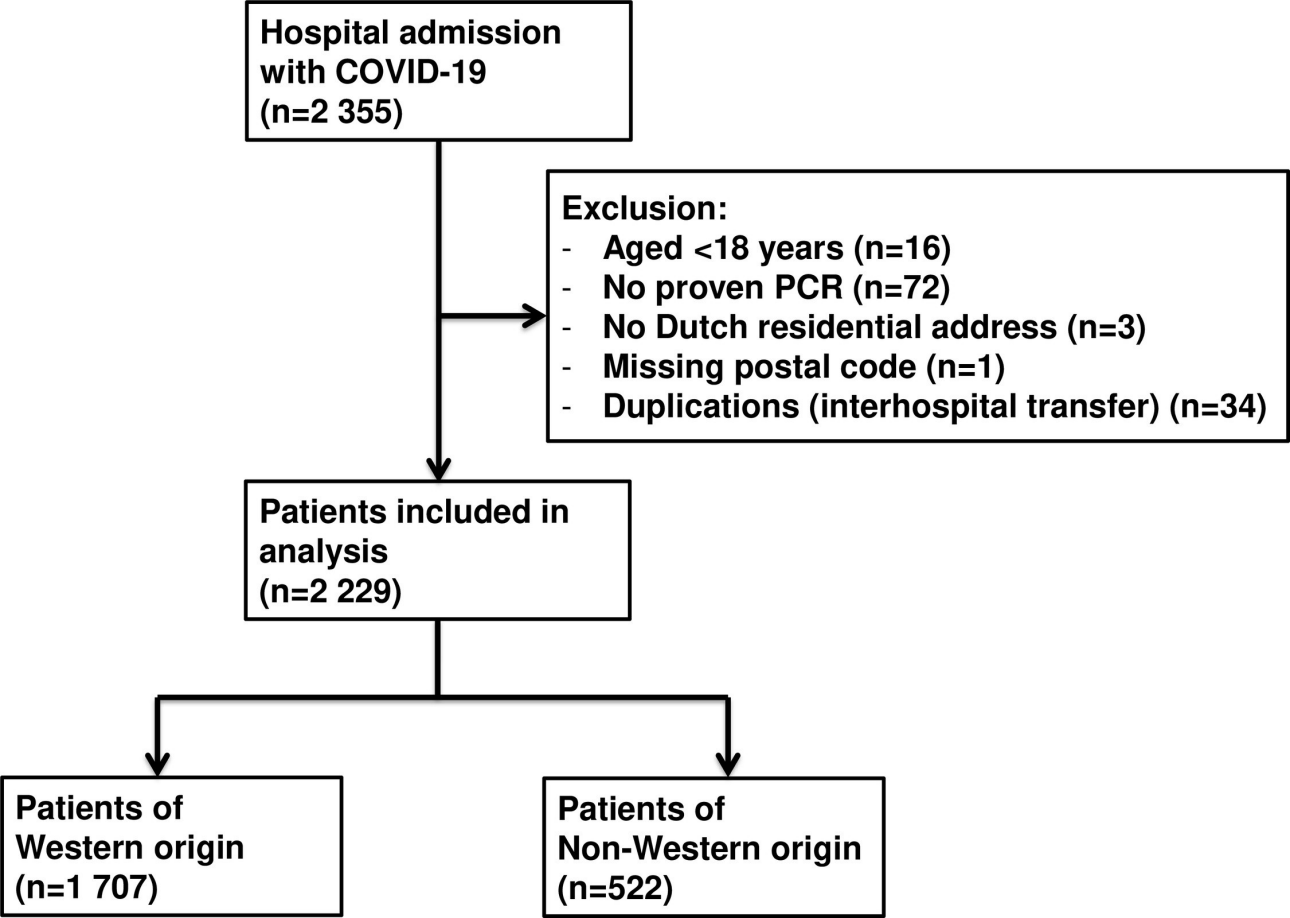
Study population flowchart.

Of the 2,355 patients, 2,229 patients met the inclusion criteria and were included in this study (Fig 1). Of the included patients, 1,707 (76.6%) were of Western origin and 522 (23.4%) were of non-Western origin. Of the 522 patients of non-Western origin, 224 were Moroccan, 105 were Turkish, 89 were Surinamese and 104 were from other non-Western origins. Table 1 shows the baseline characteristics. Non-Western persons were younger, had a lower income and lower level of education and were living in more densely populated areas than patients of Western origin. Furthermore, non-Western persons had a higher body mass index, had more often diabetes mellitus and less often cardiovascular diseases, chronic obstructive pulmonary disease and malignancies than patients of Western origin. In addition, persons of non-Western origin scored lower on the Charlson Comorbidity Index than patients of Western origin.

**Table 1:** Baseline characteristics stratified for migration background.

Patients	Total N = 2,229		Western origin N = 1,707		Non-Western origin N = 522	
Age (years) (IQR)	65.9	(56.2–76.0)	67.8	(58.7–76.8)	59.0	(48.8–70.5)
Sex, male (%)	1,305	(58.5)	1,011	(59.2)	294	(56.3)
Body mass index^a^ (kg/m^2^) (IQR)	27.7	(24.7–31.3)	27.4	(24.5–31.0)	28.6	(25.5–31.9)
Income of households						
Low	749	(33.6)	419	(24.5)	320	(63.2)
Intermediate	779	(34.9)	669	(39.2)	110	(21.1)
High	701	(31.4)	619	(36.3)	82	(15.7)
Level of education						
Low	744	(33.4)	486	(28.5)	258	(49.4)
Intermediate	749	(33.6)	619	(36.3)	130	(24.9)
High	736	(33.0)	602	(35.3)	134	(25.7)
Population density						
High	745	(33.4)	418	(24.5)	327	(62.6)
Intermediate	745	(33.4)	615	(36.0)	130	(24.9)
Low	739	(33.2)	674	(39.5)	65	(12.5)
Charlson Comorbidity Index (IQR)	3	(1–5)	3	(2–5)	2	(1–4)
Cardiovascular disease (%)	579	(26.0)	485	(28.4)	94	(18.0)
Diabetes mellitus (%)	553	(24.8)	351	(20.6)	202	(38.7)
COPD (%)	248	(11.1)	225	(13.2)	23	(4.4)
Malignancy (%)	328	(14.7)	290	(17.0)	38	(7.3)
Hypertension (%)	988	(44.3)	782	(45.8)	206	(39.5)
Chronic use of systemic corticosteroids (%)	189	(8.5)	157	(9.2)	32	(6.1)

SES, socioeconomic status; COPD, chronic obstructive pulmonary disease.

^a^Body mass index missing in 179 patients

The median follow-up was 7 (IQR 4–13) days. Follow-up ended for 1,697 (76.1%) patients with discharge from the hospital, 313 (14.0%) patients died during admission, 140 (6.3%) patients were transferred to another hospital and 79 (3.5%) patients were still admitted at the end of follow-up.

### Migration background of the general population and hospitalized patients

In the general population of the province of Utrecht in the Netherlands (source population), 1,160,618 persons had a Western origin and 194,216 were of non-Western origin in 2020 (Table 2). Of the non-Western persons, 30% were Moroccan, 16% were Turkish, 10% Surinamese and 45% had another non-Western origin. As compared with persons with a Western origin, the OR for non-Western persons was 1.8 (95% CI 1.7–2.0) for hospitalization with an OR of 2.6 (95% CI 2.3–3.0) for Moroccan subjects, an OR of 2.3 (95% CI 1.9–2.8) for Turkish subjects and an OR of 3.1 (95% CI 2.5–3.9) for Surinamese subjects. The OR for Non-Western persons as compared with the Western population was 2.1 (95% CI 1.7–2.5) for ICU admission. The OR for ICU admission was 2.9 (95% CI 2.1–3.8) for Moroccan subjects, 2.4 (95% CI 1.6–3.7) for Turkish subjects and 3.5 (95% CI 2.3–5.4) for Surinamese subjects. For in hospital death, ORs were also increased for non-Western persons (OR 1.3 (95% CI 1.0–1.7)), including Moroccan subjects (OR 2.1 (95% CI 1.4–3.1)), Turkish subjects (OR 1.6 (95% CI 1.0–2.9)) and Surinamese subjects (OR 3.0 (95% CI 1.7–5.3)). We did not find increased risks of hospitalization, ICU admission or in-hospital mortality in non-Western persons other than Moroccan, Turkish or Surinamese subjects.

**Table 2:** Association between migration background and COVID-19 related hospital admission, ICU admission and mortality.

		General population	Hospital Admission	ICU Admission	Mortality	Hospital Admission		ICU admission		Mortality	
		N = 1,354,834	N = 2,229	N = 503	N = 313	OR (95%CI)		OR (95%CI)		OR (95%CI)	
Western		1,160,618	1,707	374	258	1	(Ref)	1	(Ref)	1	(Ref)
Non-Western		194,216	522	129	55	1.8	(1.7–2.0)	2.1	(1.7–2.5)	1.3	(1.0–1.7)
	Moroccan	57,563	224	53	27	2.6	(2.3–3.0)	2.9	(2.1–3.8)	2.1	(1.4–3.1)
	Turkish	30,783	105	24	11	2.3	(1.9–2.8)	2.4	(1.6–3.7)	1.6	(1.0–2.9)
	Surinamese	19,441	89	22	13	3.1	(2.5–3.9)	3.5	(2.3–5.4)	3.0	(1.7–5.3)
	Other	86,429	104	30	4	0.8	(0.7–1.1)	1.1	(0.7–1.6)	0.2	(0.1–0.6)

ICU, intensive care unit; AKI, OR, odds ratio; CI, confidence interval.

### Migration background and mortality among hospitalized patients

There were 313 in-hospital deaths, of which 258 among patients of western origin and 55 among non-Western persons (Table 3). Non-Western background was not associated with an increased HR as compared with persons with a Western origin (HR 0,7, 95% CI 0.5–1.1). After adjustment for explanatory variables including age, sex, body mass index, hypertension, Charlson Comorbidity Index, chronic use of systemic corticosteroids before admission, environmental socioeconomic status and population density, the HR was 0.9 (95% CI 0.7–1.3) for non-Western hospitalized persons as compared with hospitalized patients of Western origin. There was no association between the subcategories of patients of non-Western origin and the risk of in-hospital death. Adjusted HRs for patients of Moroccan, Turkish and Surinamese origin were 0.8 (95% CI 0.5–1.2), 1.1 (95% CI 0.6–2.2) and 1.5 (95% CI 0.9–2.7), respectively.

**Table 3:** Association between migration background and in-hospital death.

			Number of events	HR (95%CI)									
				Crude		^a^Model 1 Adjusted		^b^Model 2 Adjusted		^c^Model 3 Adjusted		^d^Model 4 Adjusted	
Western origin		N = 1,707	258	1	(ref)	1	1 (ref)	1	1 (ref)	1	1 (ref)	1	1 (ref)
Non-western origin		N = 522	55	0.7	(0.5–1.1)	1.0	(0.7–1.3)	1.0	(0.7–1.3)	1.0	(0.7–1.4)	0.9	(0.7–1.3)
	Moroccan	N = 224	27	0.8	(0.5–1.2)	0.9	(0.6–1.3)	0.9	(0.6–1.3)	0.8	(0.6–1.3)	0.8	(0.5–1.2)
	Turkish	N = 105	11	0.8	(0.4–1.5)	1.3	(0.7–2.3)	1.2	(0.7–2.3)	1.3	(0.7–2.3)	1.1	(0.6–2.2)
	Surinamese	N = 89	13	1.1	(0.6–2.0)	1.8	(0.9–3.1)	1.6	(0.9–2.9)	1.7	(0.9–2.9)	1.5	(0.9–2.7)
	Other	N = 104	4	0.2	(0.1–0.6)	0.4	(0.1–1.1)	0.5	(0.2–1.3)	0.5	(0.2–1.3)	0.4	(0.1–1.2)

HR, hazard ratio; CI, confidence interval.

^a^Model 1 Adjusted: Hazard ratio adjusted for age and sex.

^b^Model 2 Adjusted: Hazard ratio adjusted for age, sex, body mass index, hypertension, Charlson Comorbidity Index and use of systemic corticosteroids.

^c^Model 3 Adjusted: Hazard ratio adjusted for age, sex, body mass index, hypertension, Charlson Comorbidity Index, use of systemic corticosteroids and environmental socioeconomic status (proportion of minimum income households and proportion with low level of education).

* ^d^Model 4 Adjusted: Hazard ratio adjusted for age, sex, body mass index, hypertension, Charlson Comorbidity Index, use of systemic corticosteroids, environmental socioeconomic status and population density.

### Migration background and ICU admission among hospitalized patients

Of the 503 (22.6%) patients who were admitted to the ICU, 374 were from Western and 129 were from non-Western origin (Table 4). Patients of non-Western origin did not have an increased HR of ICU admission compared with the Western reference group (adjusted HR 1.1 (95% CI 0.9–1.4)). Subgroup analysis in non-Western persons showed similar HRs.

**Table 4:** Association between migration background and ICU admission.

			Number of events	HR (95%CI)									
				Crude		^a^Model 1 Adjusted		^b^Model 2 Adjusted		^c^Model 3 Adjusted		^d^Model 4 Adjusted	
Western origin		N = 1,707	374	1	(ref)	1	1 (ref)	1	1 (ref)	1	1 (ref)	1	1 (ref)
Non-western origin		N = 522	129	1.2	(0.9–1.5)	1.1	(0.9–1.4)	1.1	(0.9–1.4)	1.1	(0.9–1.4)	1.1	(0.9–1.4)
	Moroccan	N = 224	53	1.2	(0.9–1.6)	1.1	(0.8–1.5)	1.1	(0.8–1.4)	1.1	(0.8–1.5)	1.1	(0.8–1.5)
	Turkish	N = 105	24	1.1	(0.7–1.6)	1.0	(0.6–1.5)	1.0	(0.6–1.5)	1.0	(0.6–1.5)	1.0	(0.6–1.5)
	Surinamese	N = 89	22	1.2	(0.8–1.9)	1.1	(0.7–1.8)	1.1	(0.7–1.7)	1.1	(0.7–1.8)	1.2	(0.8–1.8)
	Other	N = 104	30	1.4	(0.9–2.0)	1.3	(0.9–1.9)	1.3	(0.9–1.9)	1.3	(0.9–1.9)	1.3	(0.9–1.9)

HR, hazard ratio; CI, confidence interval.

^a^Model 1 Adjusted: Hazard ratio adjusted for age and sex.

^b^Model 2 Adjusted: Hazard ratio adjusted for age, sex, body mass index, hypertension, Charlson Comorbidity Index and use of systemic corticosteroids.

^c^Model 3 Adjusted: Hazard ratio adjusted for age, sex, body mass index, hypertension, Charlson Comorbidity Index, use of systemic corticosteroids and environmental socioeconomic status (proportion of minimum income households and proportion with low level of education).

* ^d^Model 4 Adjusted: Hazard ratio adjusted for age, sex, body mass index, hypertension, Charlson Comorbidity Index, use of systemic corticosteroids, environmental socioeconomic status and population density.

### Migration background and length of hospital stay

The median length of hospital stay was 7 days (interquartile range (IQR) 4–13). Of patients who did not stay at the ICU, the medium length of stay was 6 days (IQR 3–9). It took a medium length of 2 days (IQR 0–4) before patients were admitted at the ICU. The medium length of stay at the ICU was 15 days (IQR 8–26). No differences in length of hospital stay or ICU stay were seen between patients of Western and non-Western origin (Table 5), nor in subgroup analysis between Western and Moroccan, Turkish and Surinamese patients.

**Table 5:** Association between migration background and length of hospital stay.

			Days (IQR)		Beta Coefficients (95% confidence interval)									
					Crude		^a^Model 1 Adjusted		^b^Model 2 Adjusted		^c^Model 3 Adjusted		^d^Model 4 Adjusted	
**Length of hospital stay**														
Western origin		N = 1,707	7	(4–13)	0	(ref)	0	(ref)	0	(ref)	0	(ref)	0	(ref)
Non-western origin		N = 522	7	(4–13)	-0.5	(-1.7–0.7)	0.2	(-1.1–1.4)	0.2	(-1.0–1.5)	0.2	(-1.1–1.5)	0.4	(-1.0–1.7)
	Moroccan	N = 224	6	(3–13)	0	(-1.8–1.8)	0.5	(-1.3–2.3)	0.5	(-1.3–2.3)	0.3	(-1.6–2.1)	0.5	(-1.3–2.4)
	Turkish	N = 105	7	(4–12)	-1.7	(-4.1–0.7)	-1.2	(-3.7–1.2)	-0.9	(-3.3–1.5)	-1.0	(-3.5–1.4)	-0.7	(-3.2–1.9)
	Surinamese	N = 89	6	(4–13)	-1.7	(-4.3–0.9)	-1.2	(-3.8–1.4)	-1.1	(-3.8–1.5)	-1.2	(-3.9–1.4)	-0.9	(-3.6–1.7)
	Other	N = 104	7	(4–12)	0.9	(-1.6–3.3)	1.8	(-0.7–4.4)	1.8	(-0.7–4.4)	1.8	(-0.7–4.4)	2.0	(-0.6–4.6)
**ICU stay**														
Western origin		N = 374	15	(8–27)	0	(ref)	0	(ref)	0	(ref)	0	(ref)	0	(ref)
Non-western origin		N = 129	16	(10–25)	1.3	(-2.2–4.8)	2.2	(-1.5–5.8)	2.3	(-1.3–6.0)	2.2	(-1.6–6.0)	2.6	(-1.4–6.6)
	Moroccan	N = 53	17	(12–28)	4.3	(-0.8–9.5)	4.3	(-0.9–9.5)	4.4	(-0.8–9.6)	3.7	(-1.7–9.1)	4.4	(-1.2–9.9)
	Turkish	N = 24	14	(9–19)	-3.8	(-10.6–3.1)	-3.4	(-10.4–3.6)	-3.4	(-10.5–3.6)	-3.4	(-10.7–3.9)	-2.1	(-9.6–5.3)
	Surinamese	N = 22	16	(10–23)	-2.9	(-10.1–4.2)	-2.5	(-9.7–4.8)	-1.8	(-9.0–5.5)	-1.7	(-9.1–5.7)	-0.7	(-8.1–6.8)
	Other	N = 30	15	(6–40)	3.0	(-3.4–9.4)	4.2	(-2.4–10.8)	4.2	(-2.5–10.8)	4.3	(-2.4–11.0)	4.8	(-2.0–11.6)

^a^Model 1 Adjusted: Beta Coefficients adjusted for age and sex.

^b^Model 2 Adjusted: Beta Coefficients adjusted for age, sex, body mass index, hypertension, Charlson Comorbidity Index and use of systemic corticosteroids.

^c^Model 3 Adjusted: Beta Coefficients adjusted for age, sex, body mass index, hypertension, Charlson Comorbidity Index, use of systemic corticosteroids and environmental socioeconomic status (proportion of minimum income households and proportion with low level of education).

* ^d^Model 4 Adjusted: Beta Coefficients adjusted for age, sex, body mass index, hypertension, Charlson Comorbidity Index, use of systemic corticosteroids, environmental socioeconomic status and population density.

## Discussion

In this cohort study, the main finding was that non-Western persons, including Moroccan, Turkish and Surinamese subjects, had increased odds ratios of hospital admission, ICU admission and death related to COVID-19 when the distribution of these non-Western persons in the hospital were compared with the source population. In contrast, there were no differences when the comparison between non-Western persons and Western persons were restricted to hospitalized patients. Non-Western origin as compared with patients of Western origin among hospitalized patients was neither associated with in-hospital mortality, nor with ICU admission or length of hospital or ICU stay.

Several previous studies showed conflicting results in the association between ethnicity and mortality [1–9, 12–14, 19–21]. An important reason for the differences between studies in the mortality risk, could be differences in the source population of the studies [1–9, 12–14, 19–21]. The results are probably different when using the general population as source population instead of hospitalized patients. Indeed, a recent meta-analysis showed that in hospitalized patients in the United States [13], there was no higher risk of mortality in the ethnic minority groups than white patients. In addition, it was shown that ethnic minorities were more likely to be hospitalized than white Americans [13]. Another recent study in the Netherlands showed that the risk of COVID-19 hospitalization was higher in all ethnic minority groups compared to the Dutch, but the risk of adverse outcomes after hospitalization was similar [15]. This is in line with our finding that patients with a migration background have an increased hospitalization risk and that among hospitalized patients, there is no association between migration background and mortality.

The association between migration background and ICU admission has also been investigated by several other studies. Most of the studies describing the association between ethnicity and ICU admission originate from the United States [13]. Although several studies showed increased risks of ICU admission [5, 22], other studies did not [23, 24]. A study from Canada showed that Asian and black immigrants had a higher risk of ICU admission compared with white persons [25]. A study from the United Kingdom showed that South Asian ethnicity was associated with a reduced risk of admission to ICU [26]. In contrast, another study from the United Kingdom showed that black, Asian and other ethnic minorities had increased risks of ICU admission [27].

There are only a few studies that compared the length of hospital stay between ethnic groups [5, 28, 29]. These studies did not find an association between length of hospital stay and ethnicity, which is in line with our study. A study in the United States found similar median length of hospital stay in black (6 days) and white (7 days) subjects [5]. Furthermore, a study from the United Kingdom found no differences in the total length of hospital admission or length of ICU stay between different ethnic groups and white persons [28]. In addition, there were no differences in the length of ICU stay between white (median stay of 18 days) and non-white (median stay of 18 days) subjects in a study from an ICU population from the United States [29].

There could be several potential explanations for the differences in COVID-19 related outcomes between non-Western persons and persons of Western origin as shown in our study. Firstly, it could be that the higher prevalence of unknown underlying comorbid conditions in the non-Western persons makes this population more vulnerable for COVID-19 and adverse outcomes than the Western population [15]. Second, social and cultural differences, including scientific mistrust and adherence to COVID-19 measures and number of persons in a house-hold, could play an important role in our results [30]. Differences in vaccination between non-Western persons and persons of Western origin could not be an explanation in our study, as the inclusion period of our study ended at the beginning of the vaccination programme in the Netherlands.

The risks were not increased for non-Western subjects other than Moroccan, Turkish and Surinamese subjects. This group is diverse and includes relatively young, healthy and highly educated expatriates from non-Western countries.

We also showed that once patients were hospitalized, there were no differences in COVID-19 related outcomes between migration backgrounds. There could be several explanations for this finding. An explanation could be that in this selection of patients with severe COVID-19 requiring hospitalization, origin of patients is not critical in causing adverse COVID-19 outcomes. Another explanation could be that all hospitalized patients have the same standard quality care which could decrease differences in adverse COVID-19 outcomes between Western and non-Western persons. In addition, there could be selection bias introduced by differences in hospital presentation between Western and non-Western persons. We had no data on differences of service utilization between Western and non-Western persons.

The results of this study could have important implications for public policy. Since non-Western persons, including Moroccan, Turkish and Surinamese subjects, had increased risks of hospital admission, ICU admission and death related to COVID-19, preventive strategies (including increasing adherence to covid-19 preventive measures and increasing vaccination acceptance) targeting these high-risk groups in the general population could significantly decrease disease burden. An important step is the identification of these non-Western persons. Collecting data about migration background by health providers could help for this purpose. However, this is a sensitive political issue in many European countries. Another important aspect is to develop and provide adapted educational materials for non-Western persons. Furthermore, trusted local sources should be involved to deliver important public health messages. Additionally, vaccination programmes adapted to the needs of these non-Western persons could help to improve vaccination rates as preventive strategy. Future studies should investigate the effectiveness of preventive strategies in non-Western persons.

The major strengths of the present study are the large number of patients with information on migration background, clinical data, sociodemographic data and outcome data including length of hospital stay, ICU admission and mortality. This study also has limitations. First, migration background was determined on the basis of data in the medical records. Therefore, it could be that we have misclassified patients. We grouped patients with a Western background into one group to minimalize misclassification. We believe that it is more likely that a Western background is not reported than a non-Western background. Second, socioeconomic status and population density data were derived from the postal code of the residential address as patient-reported data were not available. In the Netherlands there is no national index of deprivation, but household income and educational level are generally considered as reliable indicators. These environmental-based data have been used in previous research in the Netherlands to assess socioeconomic status [31, 32]. Third, we had no information about comorbidities and other explanatory variables of patients with COVID-19 who were not hospitalized. Therefore, we could not investigate the role of these explanatory variables in the comparison with the general population. Finally, small risk differences between subgroups of non-Western persons might have been missed due to insufficient power.

In conclusion, non-Western persons, including Moroccan, Turkish and Surinamese subjects, had increased risks of hospital admission, ICU admission and in-hospital death related to COVID-19 on a population level. In contrast, there were no differences in ICU admission and death when the comparison between non-Western persons and Western persons was restricted to hospitalized patients.
